# World No Tobacco Day — May 31, 2015

**Published:** 2015-05-29

Each year, the tobacco epidemic kills an estimated 6 million persons worldwide, including about 600,000 who die because of secondhand smoke exposure. If current trends continue, this number is expected to reach 8 million deaths annually by 2030 (1).

Sponsored by the World Health Organization (WHO) and observed on May 31 each year, World No Tobacco Day highlights the health risks associated with tobacco use and encourages effective actions to reduce tobacco consumption. This year, WHO calls for international collaboration to stop the illicit trade of tobacco products (2).

Illicit tobacco trade is characterized by tax avoidance and tax evasion, such as bootlegging, counterfeiting, and smuggling. This practice undermines tobacco use prevention and control by increasing the accessibility and affordability of tobacco products and can reduce government tax revenue (3). An estimated one in 10 cigarettes consumed worldwide and 8%–21% of those consumed in the United States are illicit (2,4). Governments can adopt a range of measures to reduce illicit tobacco trade, as described by the WHO *Protocol to Eliminate Illicit Trade in Tobacco Products* (3).
